# The moisture distribution in wall-to-floor thermal bridges and its influence on mould growth

**DOI:** 10.14324/111.444/ucloe.000042

**Published:** 2022-10-27

**Authors:** Yucong Xue, Yifan Fan, Jiang Lu, Jian Ge

**Affiliations:** 1 College of Civil Engineering and Architecture, Zhejiang University, Hangzhou, China; 2 Center of Balance Architecture, Zhejiang University, Hangzhou, China; 3 International Research Center for Green Building and Low-Carbon City, International Campus, Zhejiang University, Haining, China; 4 School of Civil Engineering and Architecture, Zhejiang University of Science and Technology, Hangzhou, China

**Keywords:** coupled heat and moisture transfer, wall-to-floor thermal bridge, moisture distribution, mould growth

## Abstract

Moisture in building envelopes increases the energy consumption of buildings and induces mould growth, which may be amplified within the area of thermal bridges due to their different hygrothermal properties and complex structures. In this study, we aimed to (1) reveal the moisture distribution in the typical thermal bridge (i.e., wall-to-floor thermal bridge, WFTB) and its surrounding area and (2) investigate the mould growth in a building envelope that includes both a WFTB and the main part of a wall, in a humid and hot summer/cold winter region of China (Hangzhou City). The transient numerical simulations which lasted for 5 years were performed to model the moisture distribution. Simulated results indicate that the moisture distribution presents significant seasonal and spatial differences due to the WFTB. The areas where moisture accumulates have a higher risk of mould growth. The thermal insulation layer laid on the exterior surface of a WFTB can reduce the overall humidity while uneven moisture distribution may promote mould growth and water vapour condensation.

## Introduction

The moisture content in envelopes impacts energy dissipation and water condensation by modifying both heat and moisture transfer characteristics in building envelopes. Such influence becomes more obvious when building envelopes are exposed to a hot-humid climate, such as the hot summer and cold winter climate zone in China [1]. A previous study has demonstrated that the cooling, heating and yearly load are significantly underestimated when the moisture transfer is neglected, revealing the importance of the analysis of coupled heat and moisture transfer [2]. Wang et al. investigated the thermal insulation performance of a typical thermal bridge (i.e., exterior corners) under steady-state conditions [3]. The results indicate that the moisture transfer not only increases the heat dissipation of thermal bridges but also expands the influencing area [3]. Further, the phenomenon is significant with the increase in relative humidity [4].

The moisture in building envelopes not only affects the characteristics of heat transfer but also induces mould growth and the condensation of water vapour. Because the health of those living in the building could be seriously threatened when occupants, especially children, are heavily and systematically exposed to airborne fungal agents, indoor mould growth becomes an important issue with critical implications [5]. The indoor organic pollutants caused by mould can also significantly decrease the service life of building materials and components [6]. Furthermore, mould growth is not limited to the indoor surface; it can easily germinate and expand inside building envelopes, especially where high levels of humidity are likely to occur [7–11]. Unfortunately, the negative effects caused by moisture are more apparent in energy-efficient buildings due to some energy-saving methods, for example, the application of the thermal insulation layer [9]. However, the above issues can apparently be magnified by thermal bridges because the transfer process of heat and moisture in the thermal bridge area differs from that in other areas [12].

In order to eliminate the negative effects of thermal bridges, a long-term evaluation of the moisture distribution in the building envelope is urgently required. The objective of this study was to establish a model capable of simulating the coupled heat and moisture transfer (denoted as the HAMT model) in a typical thermal bridge. Among all kinds of thermal bridges that are caused by the frame structure, the wall-to-floor thermal bridge (WFTB) takes the most considerable fraction of the building envelope and has the largest heat flux [13,14]. Therefore, the building envelope with a WFTB was employed as the object in our study. The study aims to (1) display the seasonal moisture distribution of the building envelope, and (2) find high-risk areas for mould growth.

## Theoretical models

As building materials are mostly composed of not only solid matrices but also pores, moisture will be transferred in them together with heat. To describe the coupled process of heat and moisture transfer in building envelopes, the theoretical model is given in this section. On the basis of the mass conservation law, the mass balance can be written as Eq. (1), which is finally converted to Eq. (2) with Fick’s and Darcy’s laws [15].

(1) $$\frac{\partial \omega }{\partial t}=-\nabla (g_{1}+g_{\text{v}})$$

(2) $$\frac{\partial \omega }{\partial t}=\nabla \left(\left(\delta_{\text{p}}\varphi \frac{dp_{\text{sat}}}{dT}\right)\nabla T+\frac{\partial \varphi }{\partial p_{\text{c}}}\left(D_{\text{w}}\frac{\partial \omega }{\partial \varphi }+\delta_{\text{p}}p_{\text{sat}}\right)\nabla p_{\text{c}}\right)$$

where *ω* is the gravimetric moisture content (kg·m^–3^), *t* is the time coordinate (s), *g*_l_ is the liquid flux (kg·m^–2^·s^–1^), *g*_v_ is the vapour diffusion flux (kg·m^–2^·s^–1^), *δ*_p_ is the water vapour permeability (s), *φ* is the relative humidity, *p*_sat_ is the saturated water vapour pressure (Pa), *T* is the temperature (K), *p*_c_ is the capillary pressure (Pa), *D*_w_ is the liquid water diffusivity (m^2^·s^–1^), *ρ*_l_ is the density of liquid water (kg·m^–3^), g is the gravitational acceleration (m·s^–2^).

According to energy conservation law, heat conduction and enthalpy flow caused by both vapour and liquid water transfer together constitute the energy change in the controlled element, as shown in Eq. (3). By combining with Fourier’s law, the heat balance is then converted to Eq. (4).

(3) $$\frac{\partial }{\partial t}(\rho c_{\text{p}}T+h_{\text{v}}\omega_{\text{v}}+h_{1}\omega_{1})=\nabla (-q-h_{\text{v}}g_{\text{v}}-h_{1}g_{1})$$

(4) $$\rho c_{\text{p}}\frac{\partial T}{\partial t}=\nabla \left(\left(\lambda +h_{\text{lat}}\delta_{\text{p}}\varphi \frac{dp_{\text{sat}}}{dT}\right)\nabla T+\left(h_{\text{lat}}\delta_{\text{p}}p_{\text{sat}}\frac{\partial \varphi }{\partial p_{\text{c}}}\right)\nabla p_{\text{c}}\right)$$

where *ρ* is the density of the building material under the absolutely dry condition (kg·m^–3^), *c*_p_ is the specific heat capacity of the material under the absolutely dry condition (J·kg^–1^·K^–1^), *λ* is the thermal conductivity (W·m^–1^·K^–1^), *h*_lat_ is the latent heat of evaporation (J·kg^–1^).

As the WFTB is a type of linear thermal bridge, two-dimension models are suitable for our study considering both the accuracy and time efficiency. The two-dimensional formulation of Eqs. (2) and (4) can then be written as Eqs. (5) and (6).

(5) $$\frac{\partial \omega }{\partial t}=\left(\delta_{\text{p}}\varphi \frac{dp_{\text{sat}}}{dT}\right)\left(\frac{\partial^{2}T}{\partial x^{2}}+\frac{\partial^{2}T}{\partial y^{2}}\right)+\frac{\partial \varphi }{\partial p_{\text{c}}}\left(D_{\text{w}}\frac{\partial \omega }{\partial \varphi }+\delta_{\text{p}}p_{\text{sat}}\right)\left(\frac{\partial^{2}p_{\text{c}}}{\partial x^{2}}+\frac{\partial^{2}p_{\text{c}}}{\partial y^{2}}\right)$$

(6) $$\rho c_{\text{p}}\frac{\partial T}{\partial t}=\left(\lambda +h_{\text{lat}}\delta_{\text{p}}\varphi \frac{dp_{\text{sat}}}{dT}\right)\left(\frac{\partial^{2}T}{\partial x^{2}}+\frac{\partial^{2}T}{\partial y^{2}}\right)+\left(h_{\text{lat}}\delta_{\text{p}}p_{\text{sat}}\frac{\partial \varphi }{\partial p_{\text{c}}}\right)\left(\frac{\partial^{2}p_{\text{c}}}{\partial x^{2}}+\frac{\partial^{2}p_{\text{c}}}{\partial y^{2}}\right)$$

The governing equations for the HAMT model can be closed when the boundary conditions are introduced. Because both the wind-driven rain (WDR) and the convective vapour exchange cause moisture flow, Eq. (7) is used to describe the process of moisture flow from the environment to the surface of building envelopes.

(7) $$g=\beta (\varphi_{\text{amb}}p_{\text{sat,amb}}-\varphi_{\text{sur}}p_{\text{sat,sur}})+(R_{\text{WDR}}-R_{\text{runoff}})$$

where *g* is the total moisture flux (kg·m^–3^), *β* is the vapour transfer coefficient at the surface (kg·Pa^–1^·m^–2^·s^–1^), *φ*_amb_ and *φ*_sur_ are the relative humidity of the environment air and the surface, *p*_sat,amb_ and *p*_sat,sur_ are the saturation water vapour pressure of environment air and the surface (Pa), *R*_WDR_ is the moisture load caused by wind-driven rain (kg·m^–2^·s^–1^), *R*_runoff_ is the excess water that runs off at the exterior surface (kg·m^–2^·s^–1^). It is assumed that there is no splash and runoff at the exterior surface of building envelopes, all the raindrops are absorbed and the *R*_runoff_ thus equals zero [16,17]. Note that the last term on the right-hand side is zero when Eq. (7) is applied to the indoor side.

The heat flow across the surface comprises convective heat transfer, the latent heat transfer accompanied by the moisture flow, as well as the solar radiation, which is given as Eq. (8).

(8) $$q=h(T_{\text{amb}}-T_{\text{sur}})+h_{\text{lat}}\beta (\varphi_{\text{amb}}p_{\text{sat,amb}}-\varphi_{\text{sur}}p_{\text{sat,sur}})+\alpha l+(R_{\text{WDR}}-R_{\text{runoff}})c_{\text{p,l}}(T_{\text{amb}}-T_{\text{sur}})$$

where *h* is the convective heat transfer coefficient (W·m^–2^·K^–1^), *T*_amb_ and *T*_sur_ are the temperature of environment air and the surface (K), *α* is the solar absorptivity of the surface, *I* is the solar radiation (W·m^–2^). It should be noted that the third and fourth terms on the right-hand side are equal to zero when Eq. (8) is applied to the indoor side.

### Validation of the HAMT model

Because the proposed partial differential equations in the developed models are fully coupled and highly nonlinear, the commercial software COMSOL Multiphysics [5.6.0.341, COMSOL, Inc., Stockholm, Sweden] is adopted to solve the above equations simultaneously [2–4,16]. Before the HAMT model is applied, validation should be performed to ensure that the model has sufficient accuracy. The European Standard EN 15026: 2007 (Hygrothermal performance of building components and building elements) provides a normative benchmark with an analytical solution [18]. It is normally believed that the analytical solution is the exact solution for the PDEs, that is, the analytical solution can accurately describe the transfer process of heat and moisture. Therefore, whether the HAMT model fulfils some basic requirements is identified in the following.

**Figure 1:**
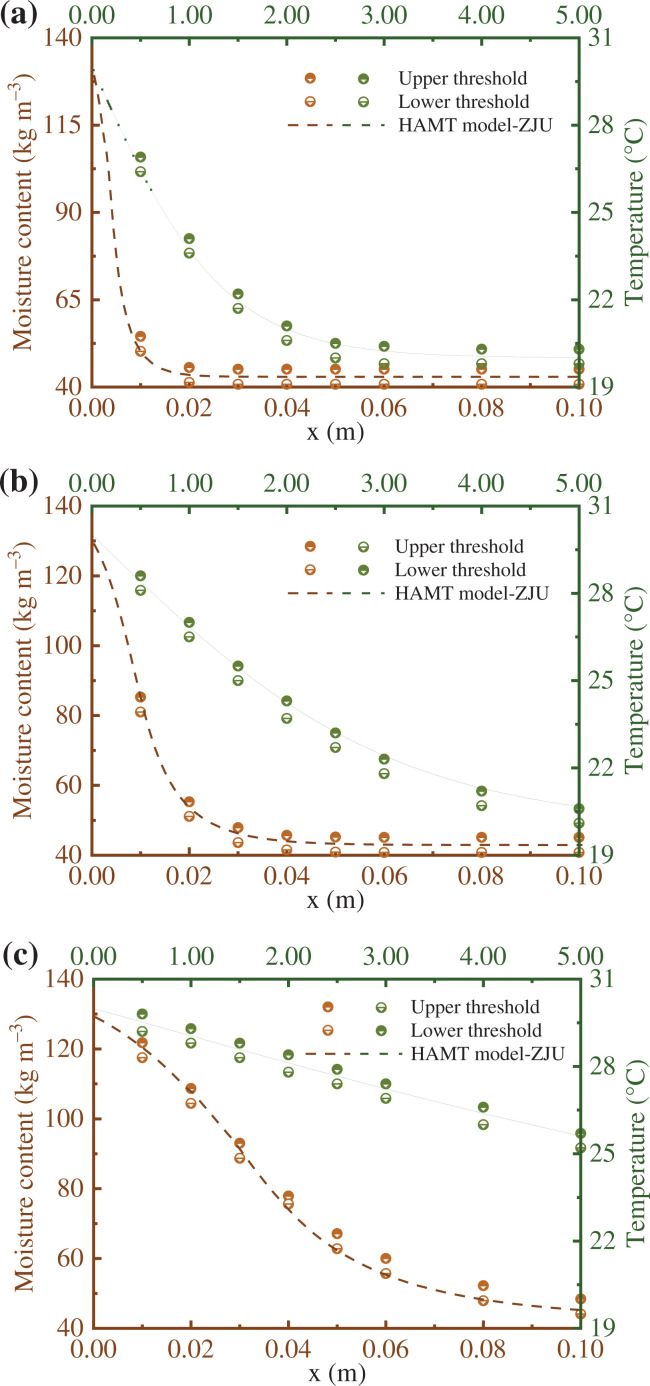
Comparison between the numerical simulation results of the HAMT model and the analytical solution of EN 15026: 2007 in temperature and moisture content profile on: (a) the 7th day, (b) the 30th day, and (c) the 365th day.

The moisture uptake in a thick single homogeneous material (semi-infinite region), which is assumed as perfectly airtight, is analysed in this benchmark. The initial condition of the material is 20°C with a relative humidity of 50%, which is in equilibrium with the surrounding environment. At a certain time, the surrounding hygrothermal environment undergoes a step change (i.e., the temperature changes to 30°C and the relative humidity changes to 95%). The material properties and other detailed descriptions are given in the European Standard EN 15026: 2007 [18]. The temperature and moisture profiles at different times are then be calculated, as shown in Fig. 1.

By comparing the numerical solution of the HAMT model with the upper/lower threshold produced by the analytical solution, it can be found that the good agreement in both temperature and moisture content is firmly proved, as Fig. 1a and b shows.

### Case settings

#### Hygrothermal properties and configurations

**Figure 2:**
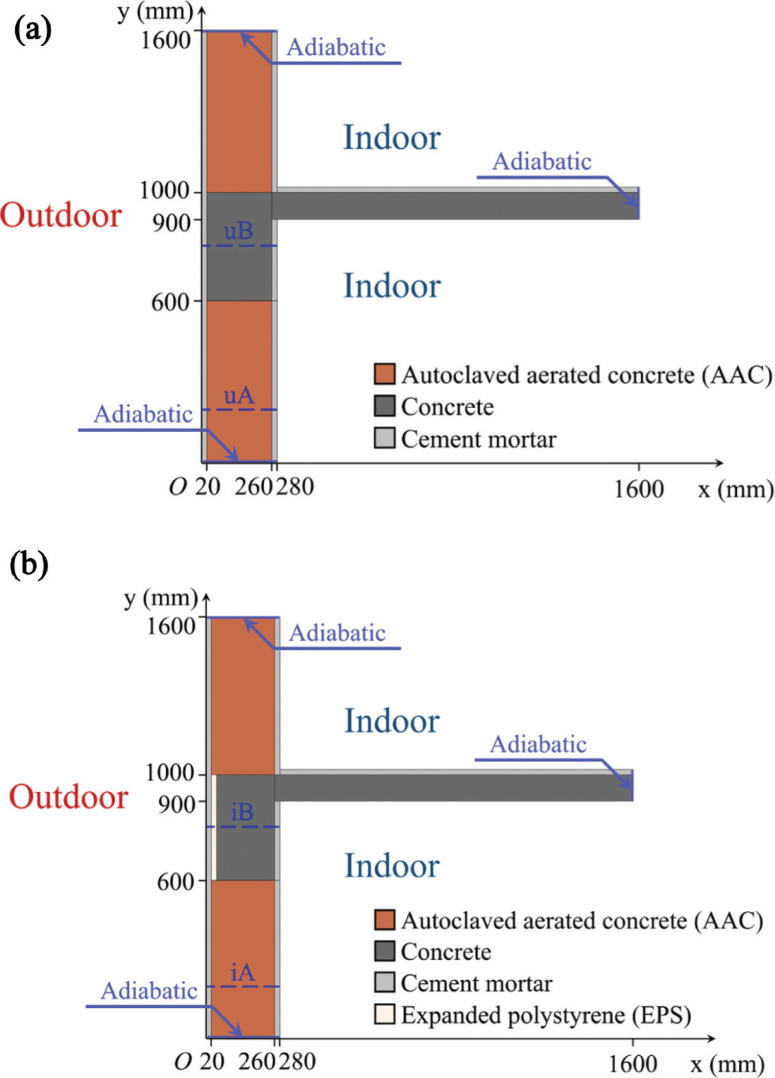
Configurations of the WFTB: (a) uninsulated, and (b) insulated.

According to the existing atlas [19–21], Fig. 2 gives two configurations of the typical WFTB that is commonly used in residential buildings in the hot summer and cold winter (HSCW) climate zone of China. The WFTB in Fig. 2a is uninsulated, whereas Fig. 2b is insulated by an additional 20 mm layer of expanded polystyrene (EPS). The cross-sections of WFTBs (see later blue arrows in Fig. 5) are set as the adiabatic boundary condition, that is, energy and mass transfer here.

Following Kumaran [22], the hygrothermal properties of the building materials are listed in Table 1. Since the building materials are assumed as homogenous, all of the hygrothermal properties are uniform in both x- and y-directions. The solar absorptance of the surface *α* is set as 0.7 [23] and the initial condition for the WFTBs is *T* = 293.15 K and *p*_c_ = 135402728 Pa.

**Table 1.** Hygrothermal properties of the building materials

		AAC	Concrete	Cement mortar	EPS
Thermal property	*ρ* (kg·m^–3^)	600	2200	1512	25
	*c*_p_ (J·kg^–1^·K^–1^)	840	940	932	1470
	*λ* (W·m^–1^·K^–1^)	*j*_1_+*j*_2_*ω*			
	*j*_1_	0.18	2.74	0.53	0.03
	*j*_2_	0.00080	0.0032	0.0031	0.0027
Hygric property	*δ*_p_ (10^–12^ s)	32.42	4.536	17.79	9.978
	*ω* (kg·m^–3^)	*k*_1_*φ*/[(1+*k*_2_*φ*)·(1–*k*_3_ *φ*)]						
	*k*_1_	91670	178.6	219.3	9.566
	*k*_2_	10690	5.971	8.731	9.237
	*k*_3_	0.9339	0.7598	0.7414	0.3788
	*D*_w_ (×10^–10^ m^2^·s^–1^)	*l*_1_·exp(*l*_2_*ω*_v_)						
	*l*_1_	0.92	0.18	27	N/A
	*l*_2_	0.0215	0.0582	0.0204	

‘*j*_1-2_’, ‘*k*_1-3_’, and ‘*l*_1-2_’ are coefficients in the equations for thermal conductivity *λ*, moisture content *ω*, and liquid diffusivity *D*_w_, respectively.

#### Background conditions

**Figure 3:**
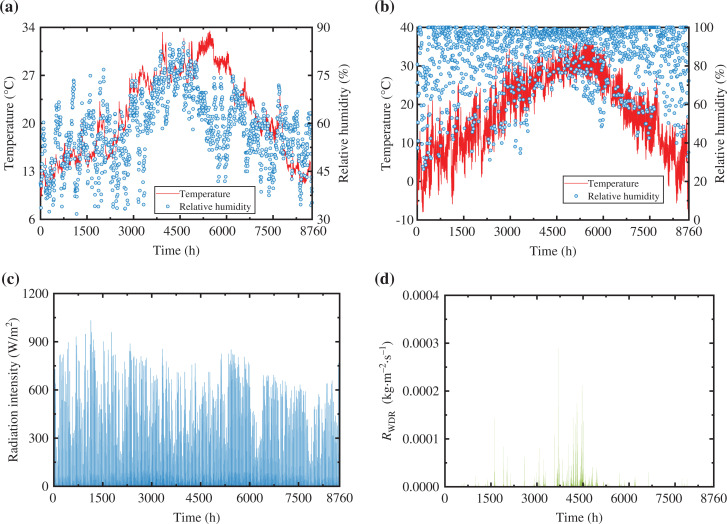
Ambient environment of WFTBs: (a) indoor temperature and relative humidity, (b) outdoor temperature and relative humidity, (c) radiation intensity *I* at the eastward vertical wall, (d) moisture load caused by wind-driven rain *R*_WDR_ at the eastward vertical wall.

A 1-year meteorological data and indoor environment data set from Hangzhou, a typical city in the HSCW climate zone, were adopted as the ambient conditions. For the indoor side, the ambient conditions (see Fig. 3a) were collected from the record of an in-situ measurement in a residential building in Hangzhou (120.1ºE, 30.3ºN). The temperature and relative humidity of indoor air at 1.1 m above the ground were real-time recorded by automatic relative humidity and temperature (RHT) recorders (JTR08ZI, JANTYTECH Co., Ltd, Beijing, China). For the outside, the meteorological data was recorded by a weather station (118.7ºE, 29.5ºN) and provided by commercial software WheatA (1.3.4, Xiaomaiya, Inc., Ningbo, China). This group of data include temperature and relative humidity, global horizontal radiation, rainfall rate on a horizontal surface, and the speed and direction of the wind (see Fig. 3b-d). On the basis of the meteorological data, our seasons can then be divided by using the methods of meteorology and climatology: spring (from 1 March to 31 May, i.e., 1417–3624 h), summer, known as the cooling season (from 1 June to 15 September, i.e., 3625–6192 h), autumn (from 16 September to 15 November, i.e., 6193–7632 h), and winter, the heating season (from 16 November to 28 May, i.e., 7633–8760 h and 1–1416 h) [24].

### Evaluation indices

#### Average of relative humidity in building materials (φ*_ave_*)

As the environment parameters are obviously affected by the seasons, the distribution of *φ* in building envelopes has evident seasonal characteristics. Therefore, the average relative humidity in the time domain *φ*_ave,t_, which can be calculated according to Eq. (9), is proposed to evaluate the overall relative humidity of building materials during a season. With the *φ*_ave,t_ for all areas of the WFTBs, a distribution of moisture can then be drawn, which is helpful to reveal the moisture accumulation area and provide guidance for mould and anti-condensation.

(9) $$\varphi_{\text{ave,t}}=\frac{\int_{k}^{j}\varphi_{i}dt}{j-k}$$

where *φ_i_* is the relative humidity at the time *i*, *j* and *k* are the range of the time domain that is used for calculation.

Based on the *φ*_ave,t_, the average relative humidity in both time and space domains *φ*_ave,ts_ is further proposed to assess the overall moisture content in particular components, for example, the thermal insulation layer, the WFTB. The *φ*_ave,ts_ is calculated according to Eq. (10).

(10) $$\varphi_{\text{ave,ts}}=\frac{\int_{k}^{j}{\int_{n}^{m}\varphi }_{i,l}dxdt}{\left(j-k)\cdot (m-n\right)}$$

where *φ_i_*_,_*_l_* is the relative humidity at the time *i* in position *l*, *m* and *n* are the range of the space domain that is used for calculation.

Thermal resistance *R* (m^2^·K·W^–1^) is widely used to evaluate the thermal performance of building envelope. After the *φ*_ave,ts_ is calculated, the overall thermal resistance can then be figured out by using Eq. (11) and the equations in Table 1.

(11) $$R=\frac{\delta }{\lambda }$$

where *δ is* the thickness of the building envelope components (m).

#### Isopleth system for evaluation of mould risk

**Figure 4:**
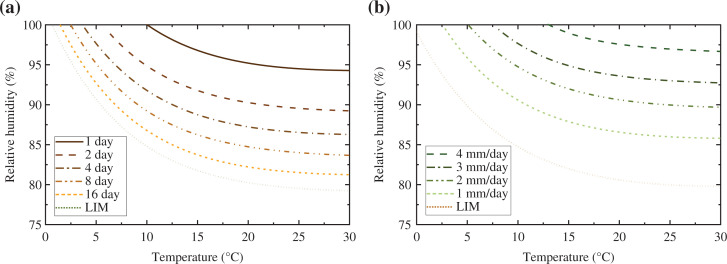
Isopleth systems for the biologically adverse recyclable building materials: (a) spore germination rate, and (b) mycelial growth rate.

In order to predict mould growth under transient boundary conditions, the Fraunhofer Institute of Building Physics (IBP) in Germany developed a bio-hygrothermal procedure, called the isopleth system [25,26]. Because the building materials (including cement mortar, concrete, expanded polystyrene, and autoclaved aerated concrete) are renderings or mineral building materials, the isopleth system for Substrate category II is therefore employed. Two groups of isolines are given by the system, one is used to predict the time required for spore germination (Fig. 4a), and the other for the evaluation of mycelial growth (Fig. 4b).

For computational calculation purposes, each isoline needs to be converted into a mathematical form. Therefore, the two-term exponential is used to fit the isolines and the fitting results are listed in Table 2. However, it should be mentioned that the isopleth system is deficient when it is used in the transient case. That is, more frequent germination of spores than the actual situation will be predicted because an interim drying out of the fungi spores cannot be taken into account.

**Table 2.** Coefficients for the equations in the isopleth system

*φ*=*a*(*e^b^*^·(^*^T^*^–273.15)^+*ce^d^*^·(^*^T^*^–273.15)^)		*a*	*b*	*c*	*d*
Spore germination	LIM	24.37	–0.1268	3.183	0.000486
	2^4^ d	24.66	–0.1265	3.219	0.000540
	2^3^ d	23.94	–0.1341	3.460	0.000163
	2^2^ d	24.61	–0.1237	3.419	0.000602
	2^1^ d	25.01	–0.1348	3.524	0.000247
	2^0^ d	26.50	–0.1205	3.447	0.000801
Mycelial growth	LIM	22.53	–0.1120	3.399	0.001034
	1 mm/d	22.26	–0.1262	3.763	0.000601
	2 mm/d	22.08	–0.1295	4.005	0.000301
	3 mm/d	23.99	–0.1526	3.852	0.000030
	4 mm/d	42.55	0.2052	2.290	–0.000297

Following Sedlbauer [25], the calculated microclimatic boundary conditions, including both temperature and relative humidity, are entered into the isopleth systems as hourly values. If the growth conditions are above the respective LIM curve (see Fig. 4) for a certain duration, mould activity may take place, which is automatically evaluated by the computer (see [25] for detailed information).

## Results

For the purpose of eliminating the influence caused by the initial value, 5-year cyclic simulations for the process of heat and moisture transfer in this study have been performed. It should be noted that only the results in the fifth year (i.e., 35,041–43,800 h), which are still numbered as 1–8760 h, are used for analysis and the corresponding calculation.

### Distribution of relative humidity

**Figure 5:**
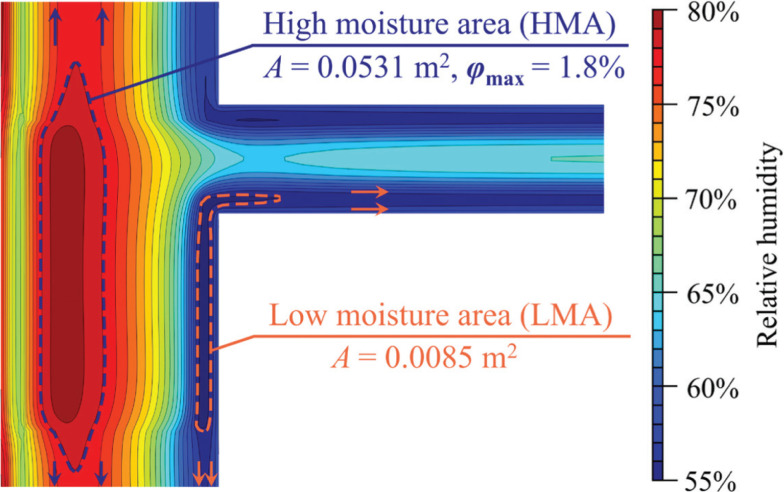
Illustration of the HMA and LMA in the insulated WFTB (the distribution of *φ* at 6057 h).

The relative humidity contours at 1% intervals are drawn in Fig. 5, in which it can be found that when the components of building envelopes are not intersecting with the others (e.g., the main part of the wall), the contours are parallel to the surface, as the arrows in Fig. 5 show. When the properties of the component change or at the corner, the contours bend and may form a closed area. Such the closed area is called the ‘high moisture area (HMA)’ when the *φ* in the closed area is higher than that of the surrounding areas, while the opposite is called the ‘low moisture area (LMA)’. In Fig. 5, the areas of the HMA (*A*) and the LMA are 0.0531 and 0.0085 m^2^, respectively. Calculating the *A* has a potential value in the practical application because it can provide guidance on the arrangement of the moisture insulation. For example, HMA confined to the range of the WFTB means that the moisture-proof measures for the WFTB alone are sufficient. Otherwise, the moisture-proof measures may need to be extended to the main part of the wall, as Fig. 5 shows. Similarly, the appearance of LMA means that it is less prone to mould growth and condensation than other adjacent areas. Furthermore, the difference (denoted as *φ*_max_) between the maximum *φ* in the HMA (*φ*_peak_) and the *φ* of the HMA boundary is 1.8%.

**Figure 6:**
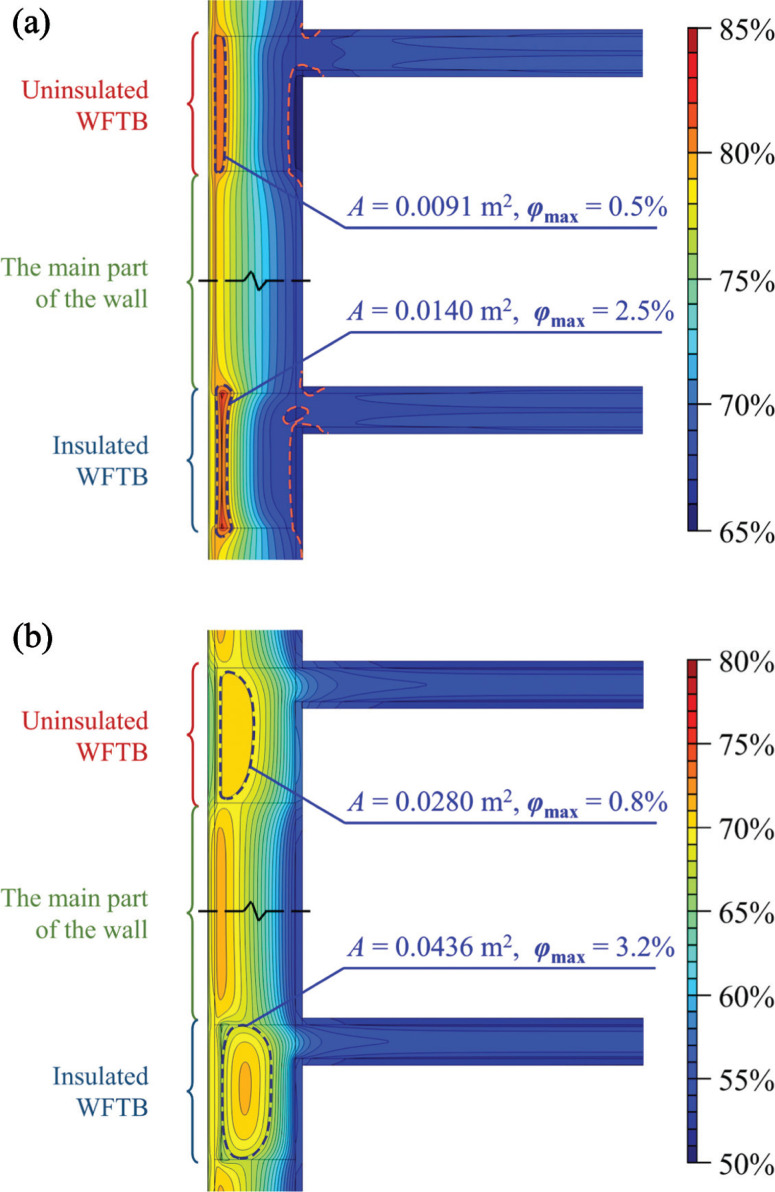
The distribution of *φ*_ave,t_ within WFTBs and their surrounding areas in (a) summer, and (b) winter.

Figure 6a and b display the distribution of *φ*_ave,t_ within WFTBs and their surrounding areas in the cooling and heating seasons, respectively. It should be noted that each figure is made up of the *φ*_ave,t_ distributions in uninsulated and insulated WFTBs to easily compare the difference in different components.

Because the relative humidity of ambient air is higher during the summer, the moisture content in WFTB is higher during the cooling season than that during the heating season. As a result, the *φ*_ave,ts_s of the uninsulated WFTB are 72.5% and 67.0% in the summer and winter, respectively; while the values of the insulated WFTB are 72.0% and 66.5%, respectively. It can be found that the *φ*_ave,ts_ of the insulated WFTB is higher than that of the uninsulated one in general. In the study case, the relative humidity of outdoor air is generally higher than that of indoor air, that is, the moisture transfers from the outside to inside most of the time. Laying an insulation layer at the exterior surface of WFTB can isolate some of the inward moisture as the insulation material (expanded polystyrene, EPS) also has a function of moisture isolation. This process finally leads to relatively low humidity in the insulated WFTB.

In the summer, by using Eqs. (10–11) and the hygrothermal properties provided by Table 1, the seasonal averages of *R* (denoted as *R*_ave,ts_) of uninsulated and insulated WFTB are calculated as 0.0824 and 0.0755 m^2^·K·W^–1^, respectively. When the season changes from summer to winter, the *R*_ave,ts_s increases only by 0.0004 and 0.0005 m^2^·K·W^–1^, respectively, which means that the change of seasons has a very limited effect on the thermal performance. However, the thermal conductivity (*λ*) of the concrete is 2.74 W·m^–1^·K^–1^ under the absolutely dry conditions, that is, the *R*s of the uninsulated and insulated WFTB are 0.0876 and 0.0803 m^2^·K·W^–1^, respectively. Therefore, the *R*_ave,ts_ of WFTB are reduced by 5.3% to 6.0%, which indicates the thermal insulation performance is obviously deteriorated due to the moisture.

The HMAs are reported in all four WFTBs in Fig. 6. Even though the overall relative humidity in the insulated WFTB is lower than that in the uninsulated one, the phenomenon of HMA is much more pronounced in the insulated WFTB, which can be reflected by a broader area (i.e., *A*) and a larger gradient (i.e., *φ*_max_). Therefore, it can be said that the adoption of an insulation layer makes the moisture distribution more uneven, which may improve the risk of vapour condensation. Another noteworthy phenomenon is that the LMAs only appear at the interior surface of the WFTBs area during the cooling season, as Fig. 6a shows. This may reduce the risk of mould growth.

### The mycelial growth in building envelopes

When the temperature and relative humidity is appropriate (see Fig. 4a), mould spores will germinate at the surface or inside the building envelopes [7,9–11]. After the spore germination, the mycelial growth will continue if the hygrothermal environment is still within an acceptable range. It should be noted that the bacteriostatic effect of sunlight was not taken into consideration in this study.

**Figure 7:**
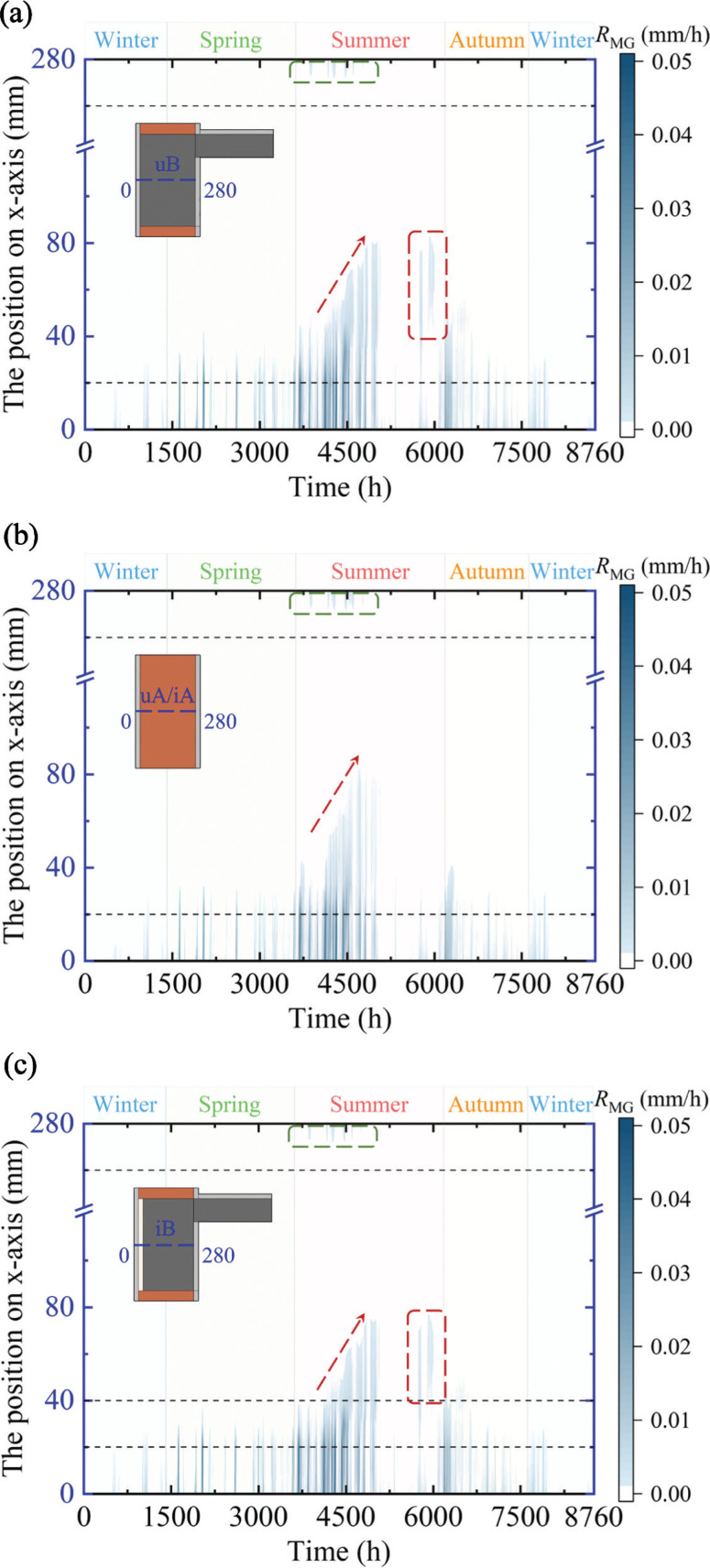
The growth rate of mycelia within different areas of building envelopes at all times of the year: (a) the uninsulated WFTB, (b) the main part of the wall, and (c) the insulated WFTB.

Figure 7 gives the rate of mycelial growth (*R*_MG_) along different cross-sections (see Fig. 2 and the illustrations in Fig. 7) in a year. The uninsulated and insulated WFTBs are represented by cross-sections uB and iB, respectively. While both cross-section uA and cross-section iA represent the main part of the wall as this area is beyond the influence range of WFTBs. Further, different chromas of the colour in Fig. 7 represent different growth rates of mycelia, that is, the darker the higher the growth rate, whereas the lighter the lower the growth rate.

Because of the relatively high relative humidity of the environment with a suitable temperature, the most suitable period for mycelial growth is early summer, that is, the plum rain season (from 29 May to 17 July, 3553–4751 h). Another period during which mycelia grow rapidly is when summer moves to autumn, which is called ‘White Dew’ to ‘Cold Dew’ (from 7 September to 8 October, 5977–6744 h) in China’s 24 solar terms. White Dew, the 15th solar term of the year, indicates the real beginning of the cool autumn. At night during this period, the temperature decreases gradually and the vapour in the air often condenses into dew, which looks white, on the foliage of plants or the exterior surface of buildings. Different from White Dew, which means the weather transitions from hot to cool, Cold Dew, as the 17th solar term in late autumn, indicates that the weather transitions from cool to cold. At this time, the air temperature is much lower than during the White Dew in most areas of China, and the dew is becoming frost. Therefore, the water vapour in the air condenses easily between these two solar terms due to the considerable diurnal temperature variation, which promotes spore germination and mycelial growth.

Besides the seasons, which have an effect on mould because of the changes in temperature and relative humidity, mycelial growth could also be affected by the moisture distribution. Under the selected indoor and outdoor boundary conditions, the moisture transfers from outside to inside in general, which results in the mould at the exterior surface growing earlier and faster than that inside the building envelopes, as the red arrows show in Fig. 7. The red boxes in Fig. 7 show a phenomenon that does not conform to the above-mentioned growth trend, that is, the mould grows only inside the building envelope and the surface part has less risk of mould growth. This phenomenon can be attributed to the existence of HMA (see Fig. 6a), which abnormally increased the relative humidity inside the building envelope and thus creates an appropriate microclimate for mould growth. Moreover, the LMAs at the interior surfaces decrease the relative humidity and create conditions that are not conducive to mould growth (see green boxes in Fig. 7). As a result, the *R*_MG_ at the interior surface is higher within the wall area than that within the WFTB area, even though this phenomenon is not as apparent as the above one.

## Discussion

The findings given by this study can enhance our understanding of how the moisture is distributed in building envelopes that include both WFTBs and the main part of the wall. It is obvious that laying a thermal insulation material outside the thermal bridge is beneficial to reduce the heat dissipation of the thermal bridges. This is not only because of the thermal isolating effect of such materials but also their function as a moisture barrier. The former can directly improve the thermal resistance of building envelopes, while the latter can prevent the thermal conductivity from increasing due to humidity by keeping the building envelopes dry. However, the application of exterior thermal insulation also causes some side effects that have not been revealed in previous studies. When the WFTB is externally insulated, the distribution of moisture content becomes more uneven, which may lead to higher risks of condensation of water vapour, spore germination, as well as mycelial growth. However, when the microclimate in building envelopes frequently switches between an appropriate condition and an inappropriate condition, mould may take a much longer time to develop or may not even react to such conditions, which is not considered in the isopleth system. Therefore, the predicted mould risk is generally higher than the real situation because of the neglect of the interim drying out of the fungi spores. In future studies, another prediction model for mould growth, such as the VTT model or biohygrothermal model [27], should be employed to further evaluate the mould risk.

**Figure 8:**
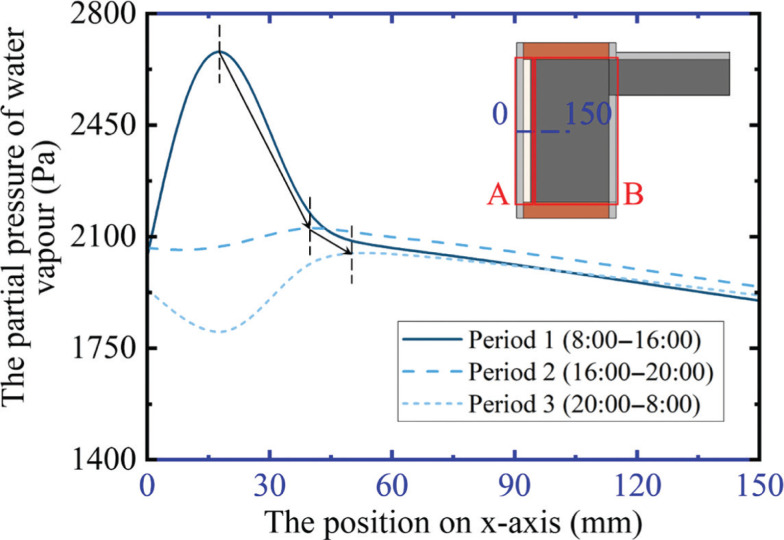
Partial pressure profile of water vapour in insulated WFTBs during winter.

Enhancing our understanding of the causes of the HMA helps to take measures to mitigate moisture accumulation. Therefore, the year-average profiles of the partial pressure of water vapour (*p*_v_) in the insulated WFTB (x = 0–150 mm, y = 800 mm) are displayed in Fig. 8. The three types of lines refer to period 1 (8:00–16:00), period 2 (16:00–20:00), and period 3 (20:00–8:00 the next day), respectively.

As Fig. 8 shows, the process of moisture accumulation can be concluded as follows: (1) in the daytime (8:00–16:00), the heat caused by the intense solar radiation is blocked in area A by the insulation layer, which results in a high temperature and low relative humidity here. Then, according to Darcy’s law, moisture transfers from the surrounding area to area A and increases the *p*_v_; (2) When the sun sets, the sharp decrease of temperature in area A raises the relative humidity. Consequently, a part of moisture follows Fick’s law and then transfers inward (as the arrows show in Fig. 8 show), leading to the increment of *p*_v_ in area B; (3) As the night wears on (20:00–8:00), the building-envelope temperature further decreases, increasing the relative humidity, and spreading the moisture in the building envelope to the ambience, which causes the *p*_v_ to decrease.

According to the above steps, the phenomenon of HMA will become more obvious when the heat flow and moisture flow increase, which means the distribution of moisture is closely related to the orientation because of the different intensity of solar radiation and moisture load caused by wind-driven rain. It is, therefore, necessary to analyse the moisture distribution in building envelopes that face different orientations in future studies. The risk of mould growth will also be obviously different when the orientation changes.

Moreover, the effect of urban heat islands (UHI) during winter could also impact the occurrence of HMA. If the UHI is more intense in period 1 (8:00–16:00) than in periods 2 and 3 (16:00–20:00 and 20:00–8:00), the HMA will become more apparent because the first step mentioned above will be enhanced and subsequently cause the building envelope to absorb much more moisture. Conversely, the HMA could be less obvious when the UHI in the last two periods is more intense. Therefore, it can be said that the UHI not only influences the energy consumption of the heating, ventilation, and air conditioning system in buildings but also has an effect on vapour condensation and mould growth.

Besides the consideration of the above factors, future studies should also propose some practical methods to alleviate the HMA for the purpose of mould proofing and condensation resistance. Moreover, the HAMT model can be further optimised for increasing the simulation accuracy, for example, considering the influence caused by air leakage. The air flows through not only the inside of the porous materials but also the interface between different materials, with which the enthalpy flow also occurs inside building envelopes and changes the energy and mass balance. As the thermal bridges alter both the temperature and relative humidity on the building surfaces, their heat transfer characteristics affect not only the energy conservation of buildings but also building thermal plumes and the environment in street canyons, which is worthy of further investigation [28,29].

## Conclusion

In order to investigate the moisture distribution and predict the mould growth risk of the insulated and uninsulated WFTBs as well as their surrounding areas, a coupled heat and moisture transfer model was developed and the process of heat and moisture transfer within the building envelopes was simulated. The average relative humidity in the time and space domain was proposed to evaluate the moisture distribution, whereas the isopleth system was adopted to predict the spore germination and mycelial growth. According to the results, the following conclusions were drawn:

1. The moisture within the WFTBs deteriorates the thermal insulation performance by 5.3–6.0%, while the seasonal variation only has a very limited influence on the thermal performance.
2. Due to the difference in hygrothermal properties of building envelope materials, there are areas where moisture accumulates (HMA) and areas where moisture disperses (LMA).
3. Laying a thermal insulation layer (EPS) at the exterior surface of WFTB reduces the overall moisture content of the WFTB. However, the moisture distribution becomes much more uneven when the EPS is used, which leads to apparent HMAs and LMAs.
4. Due to the dependence on background temperature and relative humidity, mould growth shows a significant seasonality. The distribution of moisture on the surface or inside the building envelope could also influence spore germination and mycelial growth.

In accordance with the above conclusions, the thermal insulation for building envelopes should be reconsidered when the moisture transfer is taken into account. On the one hand, the thermal insulation performance can no longer meet the requirements if the moisture transfer is considered. And, on the other hand, the application of the thermal insulation layer also brings some side effects, which have not been reported in the previous studies. Therefore, the combination of a thermal insulation layer and a vapour barrier membrane is recommended. However, it should be noted that the above conclusions were drawn for the specific case located in the HSCW climate zone and serviced by the air-conditioning system that operated in an intermittent mode, whether the conclusion could be generalised to other cases should be further discussed.

## Data Availability

Data sharing not applicable to this article as no datasets were generated or analysed during the current study.
